# Nematicidal potential of *Streptomyces antibioticus* strain M7 against *Meloidogyne incognita*

**DOI:** 10.1186/s13568-019-0894-2

**Published:** 2019-10-22

**Authors:** Manish Sharma, Shivam Jasrotia, Puja Ohri, Rajesh Kumari Manhas

**Affiliations:** 1 Department of Microbiology, Guru Nanak Dev University, Amritsar, Punjab India; 2 Department of Zoology, Guru Nanak Dev University, Amritsar, Punjab India

**Keywords:** *Meloidogyne incognita*, *Streptomyces antibioticus* strain M7, Nematicidal, Actinomycins

## Abstract

*Meloidogyne* spp. are microscopic, obligatory endoparasites with worldwide distribution which cause severe damage to agricultural crops. The present study revealed the nematicidal activity of *Streptomyces antibioticus* strain M7 against *Meloidogyne incognita*. The culture supernatant of the isolate caused 100% J2 mortality after 24 h and inhibited egg hatching (only 3%). In addition, the nematicidal activity of actinomycins V, X_2_ and D purified from strain M7 was also checked. In vitro studies displayed 97.0–99.0% juvenile mortality and 28.0–44.0% egg hatching after 168 h at 240 µg/ml of actinomycin, with LD_50_ (lethal dose) values of 28–120 µg/ml. In vivo study further validated the nematicidal activity of strain M7, where nematode infested tomato plants treated with culture supernatant/cells/solvent extract showed reduction in root galls and egg masses per plant by 50.0–62.06% and 53.48–76.74%, respectively, and significantly enhanced the shoot length (54.67–76.39%), root length (36.45–64.88%), shoot fresh weight (111–171.77%), root fresh weight (120–163.33%), shoot dry weight (54.45–145.45%), and root dry weight (100–133.3%) over the nematode infested plants treated with water. Furthermore, tomato plants treated with cells/culture supernatant/extract of strain M7 without nematode infestation also showed significant increase in various plant growth parameters. Thus, the outcome of the study revealed the potential of *S. antibioticus* strain M7 and actinomycins produced from it to be developed as safe nematicidal agents to control the root knot nematodes, and to increase the crop yield.

## Introduction

Plant-parasitic nematodes, *Meloidogyne* spp., are microscopic, obligatory endoparasites that take their nourishment from plant roots (Ruanpanun et al. 2010). They cause severe damage to plants by root gall formation and root dysfunction leading to significant yield losses of agricultural crops (Caillaud et al. 2008). These plant-parasitic nematodes have surface coat and egg shells which are considered as the major targets for biocontrol strategies (Bird and McClure 1976; Wharton 1980). The surface coat of nematodes, being the outermost layer of the cuticle, is composed mainly of proteins, chitin and lipids. Also, the nematode egg shell is composed of three layers, the outer most vitelline layer (protein), the middle chitinous layer and the inner lipid layer (Khan et al. 2004; Curtis 2008). In order to suppress *M. incognita,* the life cycle of the nematode is interrupted by volatile compounds which may be toxic to nematodes directly or they indirectly suppress nematode population by modifying the rhizosphere environment (Mishra et al. 1987). Chemical nematicides have been used to protect the plants from their infection but since these are highly toxic, expensive and become risk factor for the environment and human health, studies have been conducted to replace these chemical nematicides with naturally available biological agents (Jayakumar et al. 2005). A variety of rhizospheric microorganisms have been reported, characterized and evaluated for their biocontrol and plant growth promoting activities (El-Tarabily and Sivasithamparam 2006). Among microorganisms, actinobacteria, high G+C Gram-positive bacteria, are found to be a rich source of novel, and chemically diverse bioactive secondary metabolites with potential applications in agricultural field. *Streptomyces* spp. form the major group of actinobacteria which produce nematicidal metabolites against plant-parasitic nematodes (Dicklow et al. 1993; Samac and Kinkel 2001). *Streptomyces avermitilis,* a soil bacterium produces a mixture of avermectin B1a (> 80%) and avermectin B1b (< 20%). This bioactive compound, used under the name of Avicta and Agri-Mec, showed nematicidal activity against *Meloidogyne* spp. (*M. incognita, M. arenaria* and *M. javenica*) (Burg et al. 1979). Similarly, many researchers have documented the nematicidal potential of crude extracts and purified compounds from *Streptomyces* spp. against plant parasitic nematodes (Park et al. 2002; Yang et al. 2013; Jang et al. 2015; Kaur et al. 2016). Keeping in mind the increasing trend towards the use of microbial sources as biocontrol agents in agriculture sector, in the present study *Streptomyces antibioticus* strain M7 exhibiting antibacterial activity against drug resistant bacteria (Sharma and Manhas 2019) was evaluated for nematicidal potential against *M. incognita*.

## Materials and methods

### *Streptomyces antibioticus* strain M7

*Streptomyces antibioticus* strain M7 (MTCC 12926; GenBank accession no. KY548390) was isolated from rhizospheric soil of *Stevia rebudiana* (Sharma and Manhas 2019), maintained on starch casein nitrate agar slants at 4–8 °C in refrigerator and preserved the spores in 20% (v/v) glycerol suspension at − 20 °C.

### Nematode culture

The roots of tomato plant (*Solanum lycopersicum*) were used to recover the pure culture of *Meloidogyne incognita.* For in vitro assays, nematode egg masses were taken out from infected tomato roots which were surface sterilized with 1.5% of sodium hypochlorite (NaClO) solution with subsequent washing with distilled water (Hussey and Barker 1973). Surface sterilized eggs were used for egg hatching inhibition assay, and Baermann funnel method was used to extract second-stage juveniles (J2s) from egg masses by incubating egg masses in distilled water at 25 °C for 72–120 h (Siddiqui and Alam 1990).

### Production of actinomycins

To obtain actinomycins, fermentation was carried out for 4 days using starch casein nitrate (SCN) broth at 28 °C at 180 rpm. The culture broth was collected and centrifuged at 12,000 rpm for 10 min at 4 °C using REMI C-24PLUS centrifuge. The supernatant was used for screening nematicidal activity against *M. incognita*. For the recovery of nematicidal metabolites, the supernatant was adjusted to pH 5.0 using HCl (1 N) and extraction was done using ethyl acetate. The organic phase was separated from aqueous phase and concentrated to complete dryness under vacuum using rotary evaporator. The orange colored ethyl acetate (EtOAc) extract was subjected to silica gel chromatography, size exclusion chromatography and semi-preparative high performance liquid chromatography (HPLC) to purify the active compounds. The purified compounds were characterized as actinomycins V, X_2_ and D using various spectroscopic techniques (Sharma and Manhas 2019).

### In vitro nematicidal activity

To check the nematicidal efficacy of *S. antibioticus* strain M7, EtOAc extract and actinomycins (V, X_2_ and D) were dissolved in autoclaved water and used at different concentrations (30, 60, 120, and 240 µg/ml) against J2s and egg masses of *M. incognita* to determine the juvenile mortality and egg hatching inhibition, respectively and all experiments were verified by repeating three times.

### Juvenile mortality

To check the juvenile mortality rate, the nematode culture (200 J2s/50 µl) suspension was added to 35 mm Petri plate and treated with 2 ml of test samples viz. distilled water (negative control)/culture supernatant/EtOAc extract/actinomycins. The plates were incubated at 25 °C for 7 days with daily examination of dead juveniles. Light microscopy was used to count the live and dead juveniles on the basis of immobility and malformation characteristics when probed with a fine needle. The juvenile mortality rate was calculated using the formula i.e. (Sun et al. 2006)

$${\text{Mortality}}\left( \% \right)\, = \, 100\, \times \,{\text{dead juveniles}}/{\text{total juveniles}}.$$

### Egg hatching inhibition

The egg masses (200 eggs/50 µl) obtained from the infected tomato plants were treated with test samples (culture supernatant/EtOAc extract/actinomycins), and water was used as negative control. The Petri plates were incubated at 25 ± 3.0 °C for 168 h and daily examined to calculate the egg hatch rate using formula (Sun et al. 2006):

$${\text{Egg hatching}}\left( \% \right)\, = \, 100\, \times \,{\text{juveniles}}/\left( {{\text{egg masses}}\, + \,{\text{juveniles}}} \right).$$

### In vivo potential of *S. antibioticus* strain M7 against *M. incognita*

In vivo experiments were performed to study the efficacy of culture supernatant (CS), cells (CC) and EtOAc extract (SE) of *S. antibioticus* strain M7 under greenhouse conditions in the month of March, 2017 at 28 ± 2.0 °C. For tomato seedlings, seeds susceptible to *M. incognita* (N) (*Solanum lycopersicum* Mill. variety Pusa Ruby) were sown in sterile soil at 28 ± 2.0 °C for 2 weeks and after true leaf stage, plants were transplanted singly into pots of 8 cm diameter containing 100 g sterile soil. After 2 days, plants were given different treatments and divided into 8 groups; Group 1: 10 ml of nematode culture (100 J2s/ml of *M. incognita*), Group 2: 10 ml of nematode culture (100 J2s/ml of *M. incognita*) and 10 ml of culture supernatant obtained from 4th day old fermentation broth of *S. antibioticus* strain M7, Group 3: 10 ml of nematode culture (100 J2s/ml of *M. incognita*) and 10 ml of culture cell suspension (1 ± 10^6^ cells/ml) of *S. antibioticus* strain M7, Group 4: 10 ml of nematode culture (100 J2s/ml of *M. incognita*) and 10 ml of EtOAc extract (250 µg/ml) of *S. antibioticus* strain M7, Group 5: water (Negative control), Group 6: 10 ml of culture supernatant obtained from 4th day old fermentation broth of *S. antibioticus* strain M7, Group 7: 10 ml of culture cell suspension (1 ± 10^6^ cells/ml) of *S. antibioticus* strain M7, and Group 8: 10 ml of EtOAc extract (250 µg/ml) of *S. antibioticus* strain M7. Each treatment was replicated five times and the pots were kept in greenhouse (28 ± 2.0 °C) and watered daily. After 90 days, plants were uprooted carefully followed by washing with running tap water to remove the adhered soil.

### Statistical analysis

The data was recorded as shoot and root lengths, root galls per root, number of egg masses per plant root (in cm), fresh and dry weights of root and shoot, and statistical analysis was performed using Minitab (version 4.0) for one way analysis of variance (ANOVA) and Tukey’s post hoc test was done using ASSISTAT (7.7 beta).

## Results

**Fig. 1:**
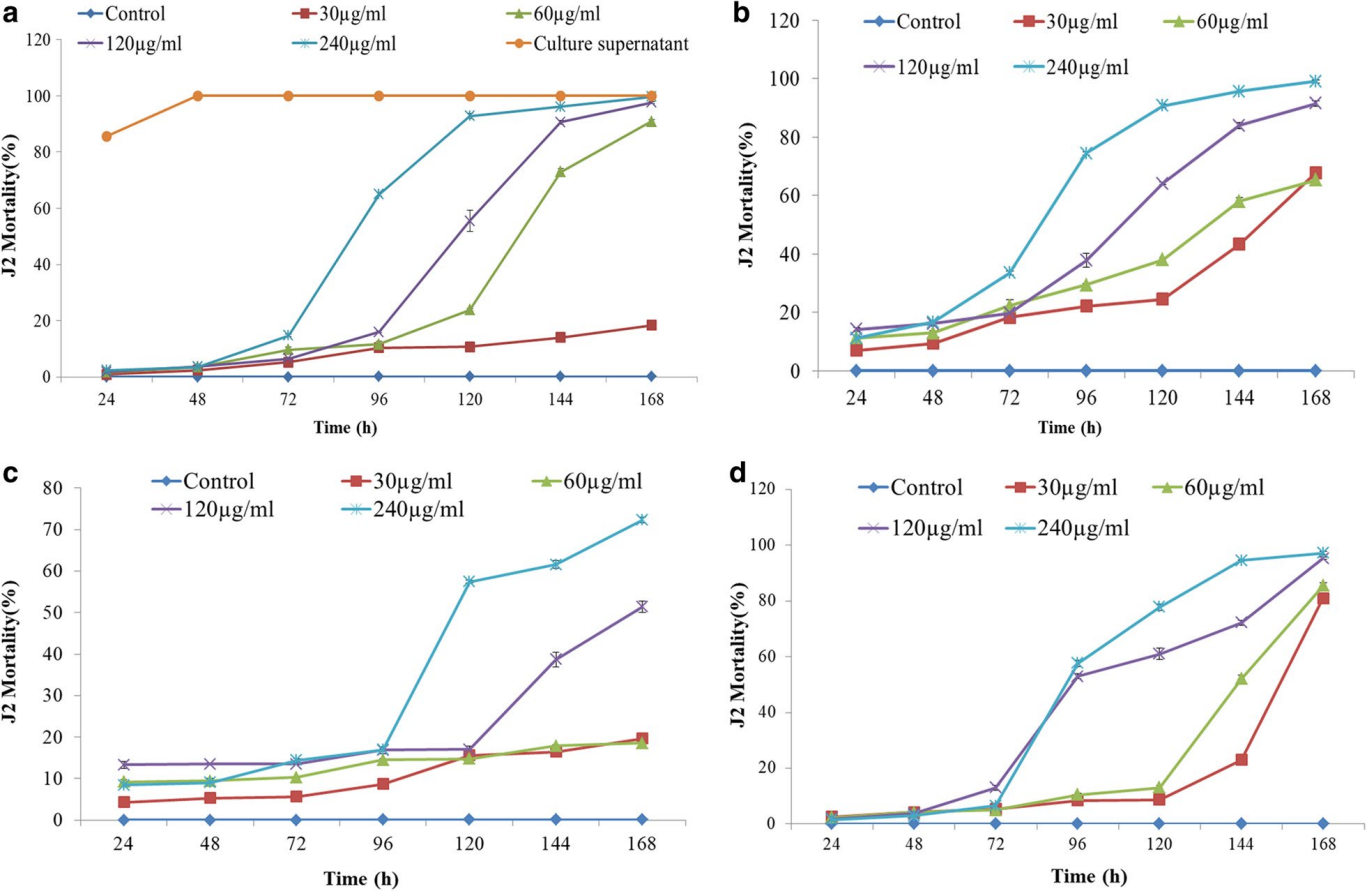
Juvenile mortality in *M. incognita* treated with *S. antibioticus* strain M7 metabolites: **a** EtOAc extract and culture supernatant, **b** actinomycin V, **c** actinomycin X_2_, **d** actinomycin D

**Fig. 2:**
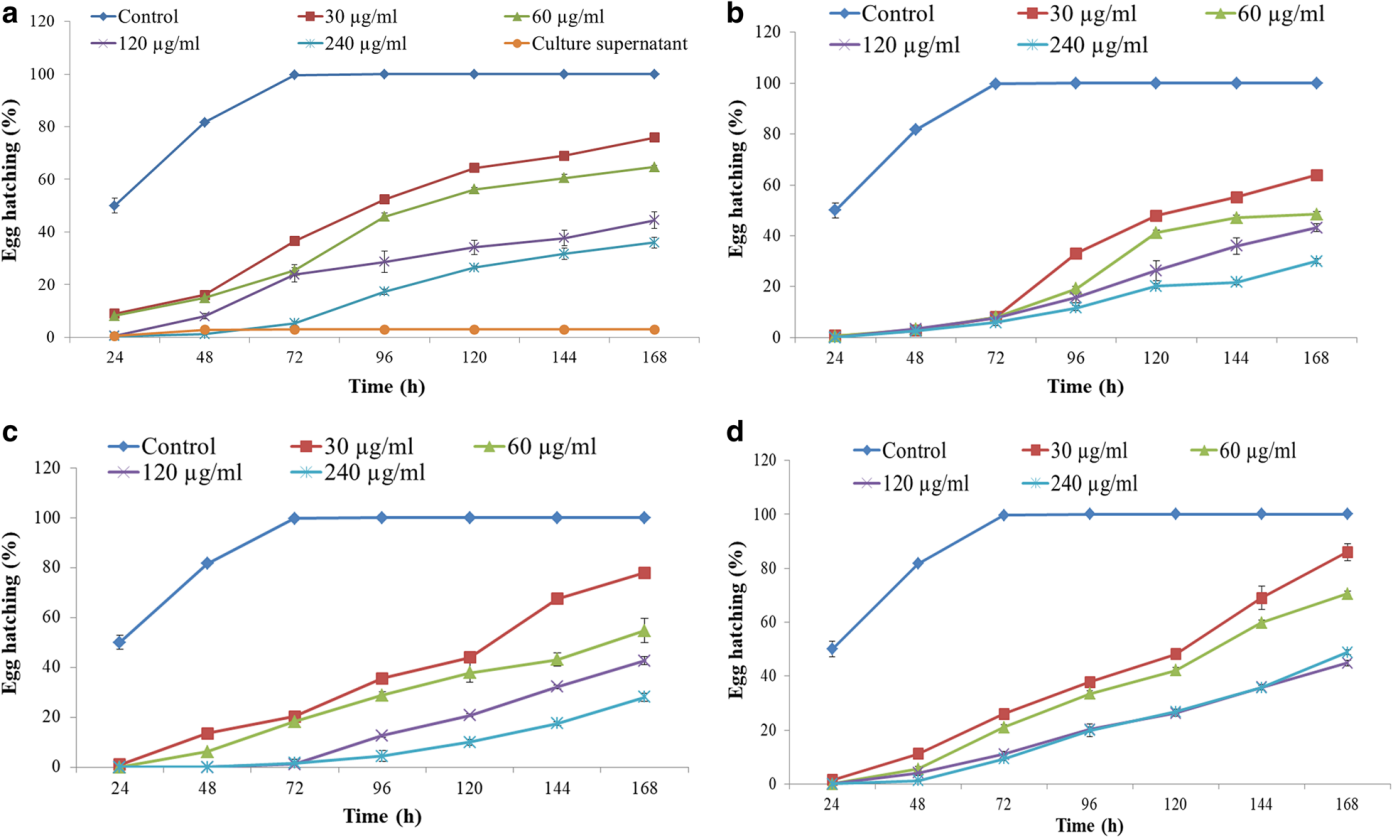
Egg hatching inhibition in *M. incognita* treated with *S. antibioticus* strain M7 metabolites: **a** EtOAc extract and culture supernatant, **b** actinomycin V, **c** actinomycin X_2_, **d** actinomycin D

*Streptomyces antibioticus* strain M7 demonstrated strong nematicidal activity against *M. incognita* (root-knot nematode). Culture supernatant (CS) and EtOAc extract (SE) of *S. antibioticus* caused significant J2s mortality and decreased egg hatching in *M. incognita* (Figs. 1a, 2a). At 24 h of incubation, 50.0% egg hatching was observed in control, whereas in case eggs treated with culture supernatant and EtOAc extract only 3% and 0.33% hatching was observed, respectively. Over the period of 168 h, the egg hatching increased to 100% in control whereas in culture supernatant egg hatching remained 3% and in presence of EtOAc extract, 36.0% egg hatching was observed at the highest tested concentration (240 µg/ml) (Fig. 2a). Similarly, culture supernatant caused 100% J2s mortality after 48 h whereas EtOAc extract resulted in the maximum of 99.66% mortality after 168 h at a concentration of 240 µg/ml (Fig. 1a).

Among the actinomycins, actinomycin X_2_ caused 99.08% juvenile mortality and reduced egg hatching to 28.0% at a concentration of 240 µg/ml after 168 h (Figs. 1b, 2b). On the other hand, actinomycin V which was less effective as compared to actinomycin X_2_, showed 72.25% juvenile mortality and 30.0% egg hatching after 168 h at a concentration of 240 µg/ml (Figs. 1c, 2c). However, when actinomycin V was evaluated at higher concentration (480 µg/ml), it showed 97% nematicidal activity after 168 h. Actinomycin D exhibited 97.16% juvenile mortality and 44.83% egg hatching (Figs. 1d, 2d.)

**Fig. 3:**
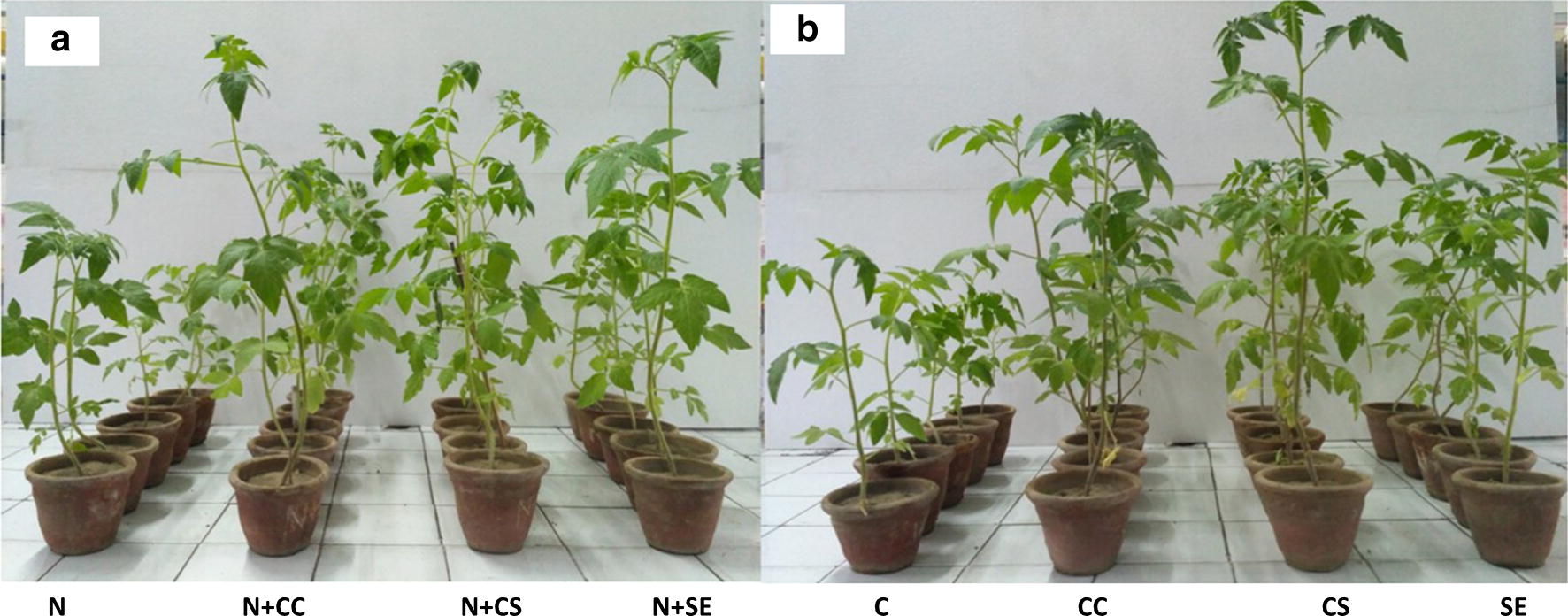
In vivo protective effect of *S. antibioticus* strain M7 and its metabolites on shoot growth of *Solanum lycopersicum* plants: **a** infested with *M. incognita* and **b** without nematode infestation: C (water), N (nematode), CC (culture cells), CS (culture supernatant), and SE (EtOAc extract)

**Fig. 4:**
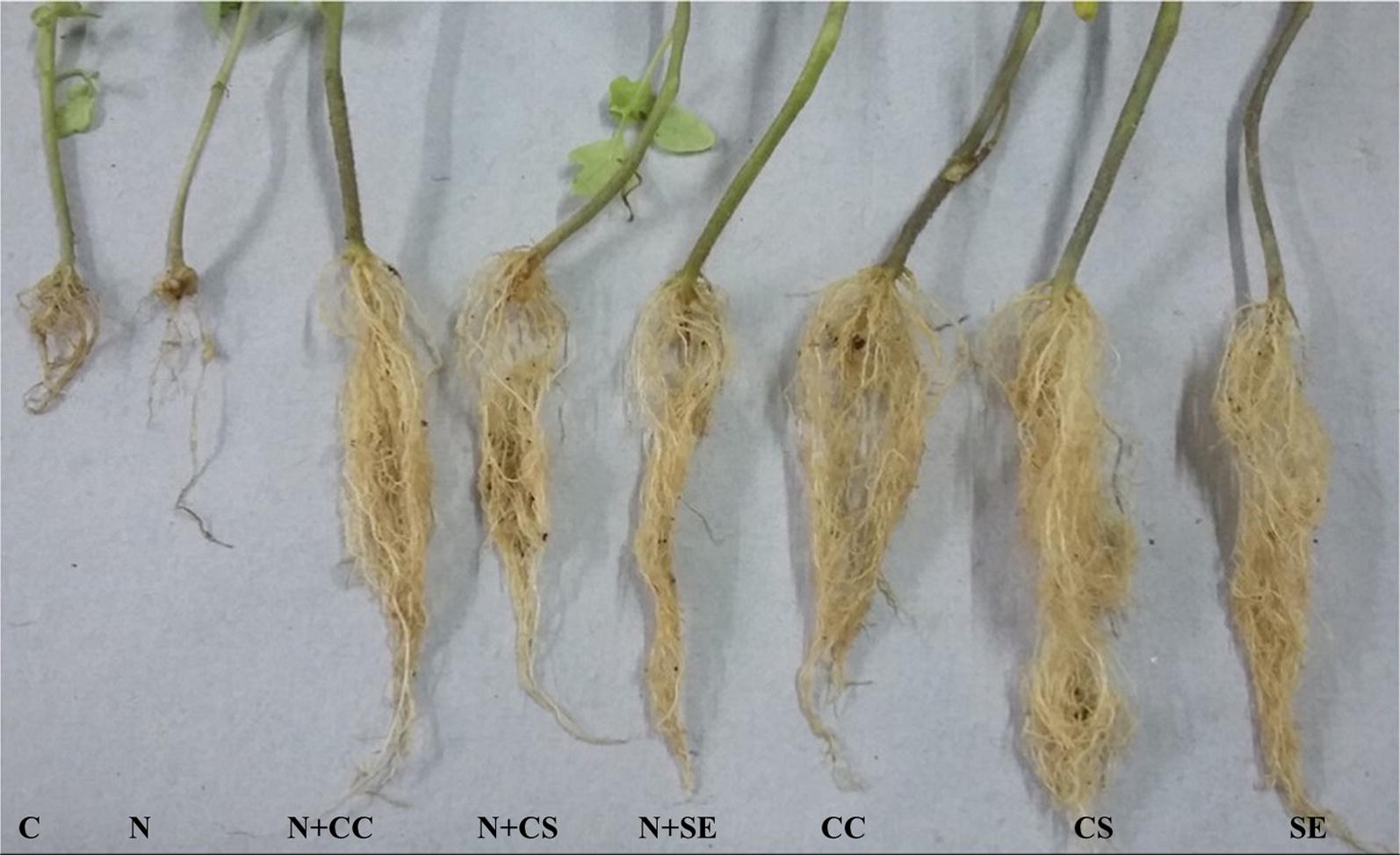
In vivo protective effect of *S. antibioticus* strain M7 and its metabolites on root growth of *Solanum lycopersicum* plants infested with *M. incognita*; C (water), N (Nematode) CC (cells of strain M7), CS (culture supernatant) and SE (EtOAc extract)

In vivo greenhouse pot experiments were carried out to further evaluate the biocontrol efficiency of strain M7. The results showed that soil drenching with culture cells, supernatant and solvent extract (EtOAc extract) in pots infested with nematode reduced the frequency of root galls and egg masses per plant by 50.0–62.06% and 53.48–76.74%, respectively. Nematode infested plants treated with culture cells also significantly enhanced the shoot length (54.67–76.39%), root length (36.45–64.88%), shoot fresh weight (111–171.77%), root fresh weight (120–163.33%), shoot dry weight (54.45–145.45%), and root dry weight (100–133.3%) (Table 1 and Figs. 3a, 4) as compared to control plants. Furthermore, in the absence of nematode stress, soil drenching with culture cells/supernatant/EtOAc extract resulted in significant increase in plant growth parameters. The treated plants showed increase of 28.25–45.80% in the shoot length, 108.33–115.59% in root length, 125.71–190% in shoot fresh weight, 563.63–690.90% in root fresh weight, 108.33–323.80% in shoot dry weight and 400–600% in root dry weight over the control plants (Table 1 and Figs. 3b, 4).

**Table 1:** Effect of *S. antibioticus* strain M7 on various growth traits of tomato seedlings infested with *M. incognita*

Treatment	Shoot length (cm)	Root length (cm)	Shoot fresh weight (g)	Root fresh weight (g)	Shoot dry weight (g)	Root dry weight (g)	No. of galls/cm root	No. of egg masses/cm root
C	28.60 ± 0.8 bc	7.44 ± 1.2 c	2.45 ± 0.07 b	0.11 ± 0.00 b	0.21 ± 0.01 b	0.02 ± 0.00 d	–	–
CC	39.08 ± 2.1 a (36.64)***	15.50 ± 2.3 ab (108.33)***	5.53 ± 0.40 a (125.71)***	0.83 ± 0.15 ab (654.54)***	0.65 ± 0.08 ab (204.52)***	0.11 ± 0.01 ab (450)***	–	–
CS	41.70 ± 3.0 a (45.80)***	15.60 ± 1.0 ab (109.67)***	6.36 ± 1.27 a (159.59)***	0.87 ± 0.34 a (690.90)***	0.89 ± 0.27 ab (323.80)***	0.14 ± 0.03 abc (600)***	–	–
SE	36.68 ± 1.8 ab (28.25)***	16.04 ± 1.6 ab (115.59)***	4.35 ± 0.24 ab (190.00)***	0.73 ± 0.10 ab (563.63)***	0.47 ± 0.05 a (108.33)***	0.10 ± 0.01 a (400)***	–	–
*M. incognita*	24.40 ± 2.3 c	10.48 ± 0.3 bc	2.09 ± 0.44 b	0.30 ± 0.04 ab	0.22 ± 0.03 ab	0.03 ± 0 cd	11.6 ± 1.2 a	8.6 ± 0.5 a
*M. incognita *+ CC	42.38 ± 1.4 a (73.68)**	17.28 ± 1.5 a (64.88)**	5.24 ± 0.34 a (150.71)**	0.79 ± 0.18 ab (163.33)**	0.42 ± 0.05 ab (90.90)**	0.06 ± 0.01 abcd (100.00)**	5.8 ± 0.3 b (50.00)*	4.0 ± 1.7 b (53.48)*
*M. incognita *+ CS	43.04 ± 2.5 a (76.39)**	14.30 ± 1.0 ab (36.45)**	5.68 ± 0.44 a (171.77)**	0.66 ± 0.08 ab (120.00)**	0.54 ± 0.04 ab (145.45)**	0.07 ± 0.01 abcd (133.33)**	4.6 ± 1.0 b (62.06)*	2.0 ± 0.8 b (76.74)*
*M. incognita *+SE	37.74 ± 2.7 ab (54.67)**	16.58 ± 1.1 ab (58.20)**	4.41 ± 0.52 ab (111.00)**	0.72 ± 0.08 ab (140.00)**	0.34 ± 0.03 b (54.54)**	0.06 ± 0.01 bcd (100.00)**	4.4 ± 0.5 b (60.34)*	3.6 ± 0.5 b (58.14)*

Mean ± SE followed by different letters with in a column are significantly different. Tukey’s test P < 0.05

*C* water, *CC* culture cells, *CS* culture supernatant, *SE* EtOAc extract

* Values indicate percentage reduction over nematode infested plants

** Values indicate percentage increase over nematode infested plants

*** All other bracketed values indicate percentage increase over control plants

## Discussion

In agriculture, use of microorganisms for the biological control offers sustainable solution to control the harmful effects of pests (Davies 2005; Davies and Spiegel 2011) and many researchers have reported different microorganisms possessing nematicidal activity (Gu et al. 2007; Huang et al. 2010). Actinobacteria are considered the most important microbial resources which produce bioactive metabolites that can inhibit or even kill nematodes (Wang et al. 2010; Begum et al. 2011; Zeng et al. 2013). *Streptomyces* spp. are the major group of actinobacteria which show activity against plant parasitic nematodes by producing extracellular enzymes and other toxic compounds. The secondary metabolites from streptomycetes have got growing interest for the development of an ecofriendly and safe integrated crop management.

The culture broth of *S. avermitilis* Manp isolate induced 89.54% J2 mortality and reduced egg hatching to 15.73% after 72 h (Jayakumar 2009). In 2016, Kaur et al. demonstrated 90% J2 mortality and 1% egg hatching after 72 h treatment with culture supernatant of *S. hydrogenans* strain DH16. In the present study, culture supernatant of *S. antibioticus* strain M7 was found to be more effective, showing 100% juvenile mortality and 3% egg hatching after 24 h and 144 h of incubation, respectively. The EtOAc extract of strain M7 showed 92.83% juvenile mortality and egg hatching of 17.33% at concentration of 240 µg/ml after 96 h. Jang et al. (2015) showed 83.5% J2 mortality in solvent extract of *Streptomyces netropsis* at concentration of 1000 µg/ml after 72 h.

In the present study, among three actinomycins produced by *Streptomyces antibioticus* strain M7, actinomycin X_2_ was more effective, showing 99.08% juvenile mortality with LD_50_ of 28.36 µg/ml after 144 h, whereas actinomycin D and V showed juvenile mortality of 97.16% and 72.25% with LD_50_ values of 60 µg/ml and 130 µg/ml, respectively. Similarly, actinomycin X_2_ decreased egg hatching to 17.50% whereas actinomycin D and actinomycin V reduced it to 35.66% and 21.66%, respectively after 144 h at concentration of 240 µg/ml. Ruanpanun et al. (2011) reported a *Streptomyces* sp. CMU-M021 producing two antifungal compounds i.e. fervenuline and isocoumarin exhibiting nematicidal activity. Out of the two compounds, isocoumarin showed weak effect on J2 mortality and no effect on egg hatching whereas fervenuline showed 100% juvenile mortality and 5% egg hatch after incubation for 96 h and 160 h, respectively at concentration of 250 µg/ml which is comparable to nematicidal activity of purified compound actinomycin X_2_ in the present study. Kaur et al. (2016) reported a novel antifungal compound from *Streptomyces hydrogenans* strain DH16 possessing strong nematicidal activity, having LD_50_ of 50 µg/ml for J2s mortality, and 14% egg hatching after 72 h of incubation at concentration 100 µg/ml.

The results of in vitro experiments showed that actinomycins exhibited significant nematicidal activity against *M. incognita,* and can be used as nematicides to control root-knot nematodes (RNKs). Actinomycins are one of the clinically important, chromopeptide lactone anticancer drugs which have been generally used for the treatment of Wilms’ tumor and childhood rhabdomyosarcoma (Chen et al. 2012). These drugs are also in use against bacterial and fungal pathogens such as *S. aureus*, *B. subtilis*, *S. faecalis*, *E. coli*, *P. aeruginosa*, *C. albicans*, *A. niger* and *A. flavus* (Hamza et al. 2013; Kulkarnia et al. 2017). Although, these drugs were discovered in 1940′s, but their biological and medicinal relevancies are still to be explored in many research areas.

In 1979, Sawhney and Webster (1979) evaluated in vivo effect of actinomycin D against *Meloidogyne incognita* and observed no gall formation in treated tomato plants. In the present study, in vivo pot experiment demonstrated positive effect of treatment of plants with culture cells/supernatant/EtOAc extract (containing actinomycins) of strain M7 on plant growth by reducing the formation of root galls and egg masses in nematode infested tomato plants. Similarly, in the absence of nematode stress, treatment of plants improved the quality of plant, showing enhanced root and shoot lengths, and root and shoot weights as compared to control plants treated with water only. The culture supernatant was found to be more effective as compared to cells and EtOAc extract, nearly eliminated root-galling and reduced egg masses per plant.

This study is the first report demonstrating the nematicidal potential of *Streptomyces antibioticus* strain M7 against *M. incognita.* The results of in vitro and in vivo studies indicate that culture cells and EtOAc extract (actinomycins) have the potential to control nematode infestations and to reduce its ill effects on crop production. Therefore, the *Streptomyces* strain M7 and its metabolites might be developed as safe biopesticide and effective fertilizer to control phytopathogens and promote plant growth.

## Data Availability

All the data and materials have been provided in main manuscript.

## Abbreviations

J2s: second-stage juveniles
SCN: starch casein nitrate
LD_50_: lethal dose
